# MUC1 gene overexpressed in breast cancer: structure and transcriptional activity of the MUC1 promoter and role of estrogen receptor alpha (ERα) in regulation of the MUC1 gene expression

**DOI:** 10.1186/1476-4598-5-57

**Published:** 2006-11-05

**Authors:** Joseph Z Zaretsky, Itay Barnea, Yael Aylon, Marat Gorivodsky, Daniel H Wreschner, Iafa Keydar

**Affiliations:** 1 Department of Cell Research and Immunology, Faculty of Life Sciences, Tel Aviv University, Ramat Aviv 69978, Israel; 2 Department of Molecular and Cell Biology, Weizmann Institute of Science, Rehovot, Israel; 3 Laboratory of Mammalian Genes and Development, Section on Transgene Regulation, NICHD, NIH, Bethesda, MD 20892, USA

## Abstract

**Background:**

The MUC1 gene encodes a mucin glycoprotein(s) which is basally expressed in most epithelial cells. In breast adenocarcinoma and a variety of epithelial tumors its transcription is dramatically upregulated. Of particular relevance to breast cancer, steroid hormones also stimulate the expression of the MUC1 gene. The MUC1 gene directs expression of several protein isoforms, which participate in many crucial cell processes. Although the MUC1 gene plays a critical role in cell physiology and pathology, little is known about its promoter organization and transcriptional regulation. The goal of this study was to provide insight into the structure and transcriptional activity of the MUC1 promoter.

**Results:**

Using TRANSFAC and TSSG soft-ware programs the transcription factor binding sites of the MUC1 promoter were analyzed and a map of transcription *cis*-elements was constructed. The effect of different MUC1 promoter regions on MUC1 gene expression was monitored. Different regions of the MUC1 promoter were analyzed for their ability to control expression of specific MUC1 isoforms. Differences in the expression of human MUC1 gene transfected into mouse cells (heterologous artificial system) compared to human cells (homologous natural system) were observed. The role of estrogen on MUC1 isoform expression in human breast cancer cells, MCF-7 and T47D, was also analyzed. It was shown for the first time that synthesis of MUC1/SEC is dependent on estrogen whereas expression of MUC1/TM did not demonstrate such dependence. Moreover, the estrogen receptor alpha, ERα, could bind *in vitro *estrogen responsive *cis*-elements, EREs, that are present in the MUC1 promoter. The potential roles of different regions of the MUC1 promoter and ER in regulation of MUC1 gene expression are discussed.

**Conclusion:**

Analysis of the structure and transcriptional activity of the MUC1 promoter performed in this study helps to better understand the mechanisms controlling transcription of the MUC1 gene. The role of different regions of the MUC1 promoter in expression of the MUC1 isoforms and possible function of ERα in this process has been established. The data obtained in this study may help in development of molecular modalities for controlled regulation of the MUC1 gene thus contributing to progress in breast cancer gene therapy.

## Background

The MUC1 gene belongs to a family of genes encoding mucin glycoproteins [1]. MUC1 is normally expressed on the apical surface of mammary epithelial cells. However, in breast adenocarcinoma and a number of epithelial tumors, MUC1 is upregulated with aberrant expression over the entire cell surface [1-4]. This characteristic makes the MUC1 protein valuable as a marker in breast cancer diagnostics and prognosis [2]. In addition to epithelial cells, expression of MUC1 glycoprotein has been observed also in hematopoetic cells, T- and B-lymphocytes, hepatocytes, myocytes and nerve cells [5,6].

Molecular studies have revealed several MUC1 protein isoforms: MUC1/TM, MUC1/SEC, MUC1/Y, MUC1/X and MUC1/Z [4,7,8]. The MUC1 isoforms demonstrate a diversity of properties and functions which may explain the crucial role of the MUC1 protein in many physiological and pathological processes. It participates in signal transduction, blastocyst implantation, epithelial cell morphogenesis, cellular adhesion, T-cell immunosuppression and matastases progression [9]. Expression of the MUC1/TM, MUC1/X, MUC1/Y and MUC1/Z is associated with the presence of malignancy, whereas expression of MUC1/SEC is observed mostly in non-malignant tissue [10].

The coordinated expression of multiple MUC1 isoforms in a wide spectrum of cells suggests a fine-tuning of mechanisms regulating MUC1 transcription in a cell- and tissue-specific manner. One of the essential steps of transcriptional regulation is binding of transcription factors to transcription *cis*-elements in promoter DNA. The combinatorial arrangement of *cis*-elements in a gene promoter is one of the important factors that determine gene transcription. For this reason, identification of *cis*-elements and their arrangement in a given promoter is of great importance and may provide clues to unravel potential mechanisms regulating transcription of a given gene.

The computer analysis of promoter DNA sequences is an appropriate method for identification of transcription *cis*-elements. Previously, we analyzed the content and composition of transcription *cis*-elements in the 3'-end 725 bp fragment of the MUC1 promoter [11]. In this relatively short fragment we found more than one hundred *cis*-elements demonstrating the high level of MUC1 promoter complexity both structurally and functionally. Nonetheless, these findings represented only a small portion of the *cis-*elements operating in the full length MUC1 promoter. One of the goals of this study was to further analyze the content and arrangement of transcription factor binding sites in the full 2872 bp MUC1 promoter and to construct a MUC1 *cis-*element map.

Many studies of transcriptional regulation have been performed by transient transfection. Usually, in such experiments, a recombinant plasmid containing a promoter (or part of it) obtained from a gene of a given type (human, for example) and coding region (test-gene) from a gene of another type (bacterial CAT-gene, luciferase gene) is used. This artificial construct is transfected into cells (mouse, for example) that naturally do not express the gene of interest. Analysis of endogenous genes in homologous systems is more biologically relevant than these artificial set-ups for several reasons. It is known that introns play important role in transcriptional regulation [12], however, in artificial systems they are excluded from this process. The results of transcription, as evaluated by RT-PCR, depend on the stability of nuclear RNA-transcripts that, in turn, is determined by the gene's 3'UTR [13]. It should be underscored that usage of transfection assays with a promoter separated from its exon-intron sequences and 3'UTR, may distort the natural mechanisms of transcriptional regulation of the given gene. The inadequacy of the described system for analysis of natural regulation of transcription becomes even more obvious when properties of transcription factors of different origins are taken into account. For instance, in certain conditions the affinity and binding specificity of mouse transcription factors for ligands and *cis*-elements may be different from those of corresponding human transcription factors or transcription factors of other non-relative species [14,15]. This may lead to aberrant transcription of a human gene in mouse cells upon transient transfection. Therefore, for obtaining more objective information on the promoter activity and transcriptional regulation, it is important to study the expression of a gene both in transiently transfected heterologous cells and in cells in which the gene of interest is expressed endogenously. In this study, we investigated the roles of different regions of the MUC1 promoter in expression of human MUC1 isoforms using transient transfection. However, in addition to ectopic transfection assays we also investigated expression of the human MUC1 gene in human epithelial cells.

MUC1 expression is predominantly hormonally regulated [16-20]. Although intensively studied [16-18], the mechanisms of steroid regulation of MUC1 transcription are still obscure. Estrogen increases MUC1 expression [17], whereas the antiestrogen ICI 164,387 inhibits estradiol-stimulated MUC1 synthesis [19]. These results imply a specific effect of estrogen and suggest that nuclear estrogen receptor(s) are likely to be involved in MUC1 transcription [19]. However, in the mouse MUC1 promoter the estrogen receptor (ER) does not bind directly to estrogen receptor *cis*-element, ERE [18]. Among the *cis-*elements found in the 725 bp fragment of the human MUC1 promoter, we have detected several sites homologous to ERE [11]. In this study of the full MUC1 promoter, we identified an additional ERE. We analyzed the role of estrogen in regulation of the MUC1 transcription in human epithelial cells *in vivo *and have investigated the potential of ER to bind *cis*-ERE of the human MUC1 promoter *in vitro*. We showed for the first time that estrogen differentially regulates expression of the MUC1 isoform specific mRNAs in human breast cancer cells: it activates expression of the MUC1/SEC isoform but does not affect expression of the MUC1/TM mRNA. By electrophoresis mobility shift assay (EMSA) we were able to demonstrate that ERα present in T47D cell lysate could directly bind to MUC1 promoter EREs, thus supporting the hypothesis that regulation of the MUC1 expression by estrogen may be realized by ER binding to EREs present in the MUC1 promoter.

## Results

### Structural analysis of the MUC1 promoter

Using computer analysis of the 2872 bp MUC1 promoter sequence, we identified multiple transcription factor specific *cis*-elements (674 sites in the sense strand and 452 site in the opposite strand) and constructed a map that shows the arrangement of these elements in the MUC1 promoter ("Additional files 1, 2, 3"). The level of homology between the MUC1 *cis*-elements in our study and corresponding consensus sequences was 0.75–1.0. This facilitated isolation of discrete *cis*-elements that were highly homologous to consensus sequences, but demonstrated a definite degree of freedom in flanking regions. The analysis of these *cis*-elements revealed several important characteristics of the MUC1 promoter:

1) The numerous *cis*-elements found in the MUC1 promoter potentially afford gene expression in a wide spectrum of cells. Among these elements were ubiquitous eukaryotic *cis*-elements as well as mammary, muscle, liver, hematopoietic and immune cell specific elements. Additionally, the MUC1 promoter contained *cis*-elements found in viral promoters. Some of the elements (Ikarus-2, AML-1 or YY1) were represented in the promoter by multiple copies, while the others (NF1-tkII or Evi F) were unique and appeared in the MUC1 promoter only once ("Additional files 1, 2, 3").

2) Although most eukaryotic promoters usually carry a single RNA-pol II initiation box (either TATA-box, GC-box or Initiator) [21-23], the MUC1 promoter carried all three motifs. Moreover, the MUC1 promoter contained numerous CAP-sites that could function as putative transcription start sites (TSS). Some of the functionally active TSS have been experimentally verified [11].

3) One important feature of the MUC1 promoter was an extensive overlapping and clustering of *cis-*elements. Overlapping of *cis-*elements could facilitate competition between different transcription factors (see "Additional files 1, 2, 3"). Competing factors may be agonistic or antagonistic, either activating or repressing transcription. For example, at position -2785/-2775 the binding site for YY1 repressor overlaps the binding site specific for transcriptional activator Pu, while in another cluster (-1432/-1407), this repressor may compete both with a repressor (Gfi-1 immunospecific repressor) and with three activators (immunospecific activator Ikarus, hepatospecific activator SREBP1 and general activator protein AP1). Thus, different arrangements of *cis*-elements in the MUC1 promoter provide conditions for competition of multiple cell- and tissue-specific transcription factors which, in turn, might determine cell and tissue specificity of MUC1 gene transcription. The construction of the *cis-*element map was the first step in the programmed study of the MUC1 transcriptional regulation. The map has been employed to design functional analysis experiments of transcription factors and their potential to compete for corresponding *cis-*elements.

### Analysis of MUC1 promoter activity by transient transfection assays

**Figure 1:**
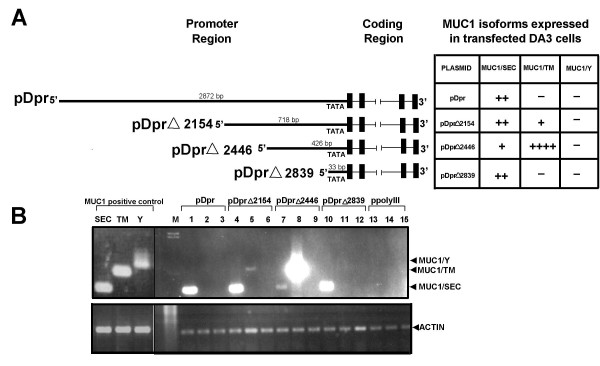
**Expression of the MUC1 isoform specific mRNA in mouse DA3 cells transfected with plasmids pDpr, pDprΔ2154, pDprΔ2446, pDprΔ2839 and ppolyIII**. DA3 cells were transfected with plasmid DNA. Cells transfected with ppolyIII were used as negative controls. DA3 cells stably transfected with MUC1/SEC, MUC1/TM and MUC1/Y cDNA were used as positive control. Total RNA was extracted 48 hrs after transfection and cDNA was synthesized. PCR amplification of MUC1 isoform specific fragments were performed using isoform specific primers. PCR products were separated by electrophoresis on 1.2% agarose gel and stained with ethidium bromide. A – Schematic structure of the pDpr, pDprΔ2154, pDprΔ2446, pDprΔ2839 plasmids and the table of the MUC1/SEC, MUC1/TM and MUC1/Y mRNA expression in transfected DA3 cells. B – RT-PCR of the MUC1 isoform specific RNA extracted from DA3 cells transiently transfected with indicated plasmids. Lane M-DNA marker; lanes 1, 4, 7, 10 and 13 (negative control) – PCR performed with MUC1/SEC specific primers; lanes 2, 5, 8, 11 and 14 (negative control) – PCR performed with MUC1/TM specific primers; lanes 3, 6, 9, 12 and 15 (negative control) – PCR performed with MUC1/Y specific primers. Positive control: DA3 cells stably transfected with MUC1 isoform specific cDNA. Lane SEC – cells expressed MUC1/SEC; lane TM – cells expressed MUC1/TM and lane Y – cells expressed MUC1/Y RNAs.

In our previous investigations [11] we employed CAT assays to evaluate the activity of a truncated human MUC1 promoter. In this study, we examined the role of different domains within the full-length MUC1 promoter in expression of MUC1 gene. The plasmid, Dpr, based on the ppolyII backbone was constructed to contain the full length (-2872/+1) human MUC1 promoter and genomic sequence of the human MUC1 gene including exons and introns. Using this plasmid we generated three sequential deletions from the 5'-end of the MUC1 promoter: pDprΔ2154 which lacks 2154 base pairs of promoter; pDprΔ2446 and pDprΔ2839 carrying only 426 and 33 residual nucleotides of the promoter sequence, respectively. These plasmids were used in transient transfection assays (Fig. 1A).

In mouse breast carcinoma DA3 cells transiently transfected with plasmid Dpr, MUC1/SEC was the only human MUC1 isoform observed (Fig 1B, lane – 1). When DprΔ2154 plasmid was expressed in mouse cells, the MUC1/TM could be detected in addition to MUC1/SEC mRNA (Fig. 1B, lanes – 4 and 5). However, the level of MUC1/TM mRNA expression was less than the expression of MUC1/SEC mRNA. Interestingly, when the plasmid DprΔ2446 that lacks an additional 292 nucleotides of the MUC1 promoter was expressed into DA3 cells, the mRNA levels of MUC1/TM dramatically increased (Fig. 1B, lane – 8) whereas the expression of MUC1/SEC isoform mRNA was much less pronounced (Fig. 1B, lane – 7). In cells over-expressing plasmid DprΔ2839, only MUC1/SEC mRNA could be detected (Fig. 1B, lane – 10). These results suggest that expression of MUC1/SEC and MUC1/TM isoform specific mRNAs in our experimental system might be controlled by different regions of the MUC1 promoter. No expression of MUC1/Y mRNA was observed in any of our transfection assays (Fig. 1B, lanes – 3, 6, 9 and 12). The reason for this is presently being studied by using different transfection and PCR parameters and changing gel running and exposure conditions.

### Expression of MUC1 isoform mRNAs in human breast cancer cell lines and the role of estrogen in transcriptional regulation of the MUC1 gene in vivo

**Figure 2:**
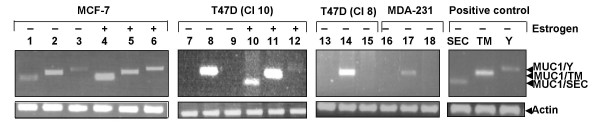
**Expression of the MUC1 isoform specific mRNA in human mammary epithelial cells detected by RT-PCR**. Total RNA from human MCF-7, T47D and MDA-231 cells and RNA from mouse DA3 cells stably transfected with MUC1/SEC (lane – SEC), MUC1/TM (lane – TM) and MUC1/Y (lane – Y) cDNA (positive controls) were extracted and RT-PCR amplification of the MUC1 isoform specific fragments were performed. Lanes 1, 4, 7, 10, 13, 16 and SEC – PCR performed with MUC1/SEC specific primers; lanes 2, 5, 8, 11, 14, 17 and TM – PCR performed with MUC1/TM specific primers; lane 3, 6, 9, 12, 15, 18 and Y – PCR performed with MUC1/Y specific primers.

The next step in our investigation was study of the MUC1 transcription in natural conditions. For this purpose we evaluated expression of the MUC1 isoform specific mRNA in human breast epithelial cells T47D, MCF7 and MDA-MB-231. We also used these cells for studying the role of estrogen and estrogen receptors (ER) in MUC1 transcription. In non-treated, ER-negative T47D (T47D, clone 8) and MDA-MB-231 cells, only MUC1/TM mRNA could be detected (Fig 2, lanes – 14 and 17, respectively). As expected, treatment of these cells with estrogen did not change the pattern of the MUC1 gene expression (data not shown). When ER-positive T47D cells (T47D, clone 10) were grown without estrogen also they expressed only MUC1/TM mRNA (Fig. 2, lane – 8). However, treatment of these cells with estrogen activated transcription of the MUC1/SEC mRNA and MUC1/Y mRNAs although the later one was expressed much less (Fig. 2, lanes – 10 and 12, respectivelly). These results imply that estrogen has a differential effect on the expression of MUC1 isoforms. Whereas the expression of MUC1/TM does not depend on estrogen, MUC1/SEC and probably MUC1/Y isoforms are expressed in an estrogen-dependent manner. In untreated MCF7 cells, both the MUC1/SEC and MUC1/TM mRNA are clearly expressed (Fig. 2, lanes – 1 and 2) but MUC1/Y mRNA was hardly detected (Fig.2, lane – 3). When MCF-7 cells were treated with estrogen, the expression of MUC1/SEC and MUC1/Y mRNAs increased (Fig. 2, lanes 4–6).

**Figure 3:**
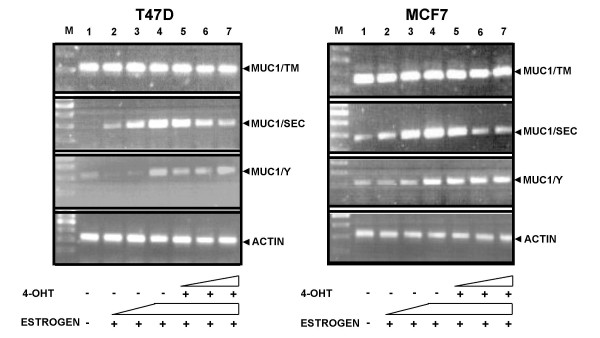
**Effect of estrogen and 4-hydroxytamoxifen (4-OHT) on expression of MUC1 isoform specific mRNA in T47D and MCF7 cells analyzed by RT-PCR**. Human T47D (clone 10) and MCF7 cells were cultured in DMEM medium with or without estrogen and 4-OHT supplements as described in "Materials and Methods". Total RNA was extracted and MUC1 isoform specific RT-PCR was performed. Lane M-DNA marker; lane 1 – cells grown in the medium without estrogen; lanes 2, 3 and 4 – cells grown with 0.1 nM, 1 nM and 10 nM estrogen, respectively; lanes 5, 6 and 7 – cells grown with 10 nM estrogen and with 10 nM, 100 nM and 1 μM 4-OHT supplements, respectively.

To clarify the role of estrogen and estrogen receptors (ER) in transcriptional regulation of the human MUC1 gene, experiments with antiestrogen 4-hydroxytamoxifen (4-OHT) were performed. 4-OHT competes with estrogen for binding to ER thus disrupting the signal transduction originating from estrogen. Fig. 3 shows that estrogen increased and 4-OHT somewhat inhibited the expression of MUC1/SEC mRNA in a dose dependent manner in both T47D and MCF7 cells (Fig. 3, lanes 2–4 and 5–7, respectively). MUC1/TM mRNA expression did not respond to these agents. Expression of MUC1/Y mRNA was not sensitive to low concentration (0.1 nM and 1 nM of estrogen (Fig. 3, lanes 2 and 3) but was increased by high concentration (10 nM) of hormone in both cell lines (Fig. 3, lane 4). In contrast to MUC1/SEC, expression of MUC1/Y was not inhibited by 4-OHT (Fig. 3, lanes 5 – 7).

Our results document the differential effect of estrogen and 4-OHT on the expression of the MUC1 isoform mRNAs. Since the above experiments suggest an involvement of ER in MUC1 transcriptional regulation at least of some of its isoforms, next we wished to analyze the binding of human ERs to the MUC1 promoter *cis*-estrogen response element (*cis*-ERE).

### Binding of ERα to cis-ERE in the MUC1 promoter

To investigate the potential binding of ER to the MUC1 *cis*-ERE we employed electrophoresis mobility shift assays (EMSAs). It has previously been shown by Western blot analysis that ERα is the main type of estrogen receptor expressed in T47D cells [24]. We confirmed this observation by RT-PCR (data not shown). Taking this into account, we concentrated our efforts on studying the ability of the MUC1 EREs to bind ERα.

Nuclear lysate of estrogen-treated T47D cells (clone 10, ER+) served as a source of ERα. The oligonucleotides utilized in this study were homologous to the six *cis*-EREs found in the MUC1 promoter by our bioinformatics search. The oligonucleotide Vit-ERE originating from the vitellogenin gene and consisting of a canonical palindrome ERE (GGTCA*NNN*TGACC) [25] was used as a positive control. It contains one direct and one inverted repeats of consensus sequence (GGTCA) separated by three intermediate nucleotides. In the MUC1 promoter, although we did not locate a classical palindrome ERE, a reminiscent *cis*-element sequence (GGACC*N*CGACC) structurally similar to Vit-ERE was identified. This *cis*-element, called *"putative" *ERE-6, was located at the position -349/-339. Five other half (1/2 of the core sequence) direct (GGTCA) or inverted (TGACC) sequences were found at positions -2748/-2744 (ERE-1), -1174/-1170 (ERE-2), -745/-741 (ERE-3), -384/-380 (ERE-4) and -364/-360 (ERE-5) (see "Additional files 1, 2, 3").

**Figure 4:**
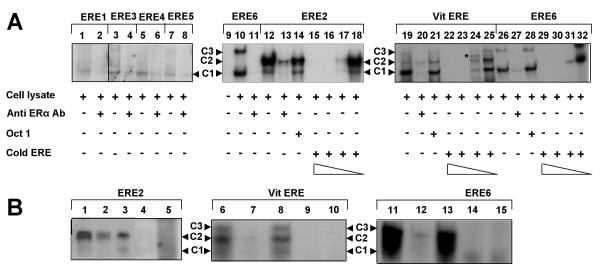
**ERα binding to EREs detected in the MUC1 promoter**. The EMSA of the complexes (C1, C2 and C3) developed by ERα from T47D nuclear lysate and ^32^P-labeled oligonucleotides containing the MUC1 promoter EREs (for details see "Materials and Methods"). **A: **Binding of ERα to ^32^P-labeled oligonucleotides ERE1, ERE2, ERE3, ERE4 and ERE5, containing 1/2 consensus core sequence of the classical ERE, and to palindrome like ERE (putative ERE-6 and Vit ERE). Lanes 1, 3, 5, 7, 10, 12, 19 and 26 – complexes developed in absence of anti-ERα Ab; lanes 2, 4, 6, 8, 11, 13, 20 and 27 – partial or complete inhibition of complex formation by anti-ERα Ab; lanes 14, 21 and 28 – binding of ERα to ^32^P-ERE2, Vit-ERE and ERE6, respectively, in the presence of "cold" Oct1 oligonucleotide; lanes 15–18 – binding of ERα to ^32^P-ERE2 in presence of decreasing amount of the "cold" ERE2; lanes 22–25 – binding of ERα to ^32^P-Vit ERE in presence of decreasing amount of the "cold" Vit ERE (* indicates an intermediate complex developed when binding reaction was kept on ice); lanes 29–32 – binding of ERα to ^32^P-ERE6 in presence of decreasing amount of the "cold" ERE6. **B: **Effects of anti-ERα Ab and normal rabbit serum (NRS) on ERα binding to MUC1 specific and mutated EREs. Lanes 1, 6 and 11 – binding of ERα in absence of anti-ERα Ab; lanes 2, 7 and 12 – partial or complete inhibition of binding complexes by anti-ERα Ab; lanes 3, 8 and 13 – binding of ERα in presence of NRS; lanes 4, 9 and 14 – binding of ERα to mutated ERE in absence of anti-ERα Ab; lanes 5, 10 and 15 – binding of ERα to mutated ERE in presence of anti-ERα Ab.

When Vit-ERE was incubated with T47D nuclear lysate, three main complexes, C1, C2 and C3, were detected (Fig. 4A, lanes 19, 21 and 25). The complex C1 had the highest gel mobility and apparently represented the simplest nucleoprotein complex composed of ERα and Vit-ERE oligonucleotide. The complexes C2 and C3, in addition to components present in the complex C1, might contain also ER-specific cofactors [26].

In our assays, all MUC1 promoter EREs could bind ERα (Fig. 4A, lanes – 1, 3, 5, 7, 10 and 12). However, the complexes developed with different ERE had different gel mobilities. The most prominent binding was observed with *"putative" *ERE-6 and ERE-2 (Fig. 4A, lanes – 10 and 12, respectively), while the affinity of ERE-1, ERE-3, ERE-4 or ERE-5 to ERα was less apparent (Fig. 4A, lanes – 1, 3, 5, and 7). The complexes containing the ERE-1, -3, -4 and -5 oligonucleotides migrated as a faint single band (Fig. 4A, lanes – 1, 3, 5, and 7) corresponding to complex "C1". The ERE-2 formed two complexes, C1 and far predominant C2, (Fig. 4A, lane – 12). The *"putative*" ERE-6 also revealed two types of complexes equivalent to C1 and C3 (Fig. 4A, lanes 10, 26 and 28). The complexes C1, C2 and C3 were observed usually when the binding reaction was kept at room temperature. However, if the binding reaction was performed on ice, an intermediate complex(es) appeared (Fig. 4A, lane 24, band*).

The specificity of ERα binding to the MUC1 promoter EREs was validated by the ability of anti-ERα antibodies to partially or completely inhibit binding ERE (Fig. 4A, lanes – 2, 4, 6, 8, 11 and 13; Fig. 4B, lanes – 2, 7 and 12). Normal rabbit serum failed to inhibit the binding of ERα (Fig. 4B, lanes – 3, 8 and 13). The mutated ERE sequences did not bind ERα (Fig. 4B, lanes – 4, 9 and 14). The electrophoretic profiles of the products obtained in reaction with mutated oligonucleotides could not be changed by anti-ERα antibodies (Fig. 4B, lanes – 5, 10 and 15). Moreover, corresponding "cold" ERE-oligonucleotides (Fig 4A, lanes 15–18, 22–25 and 29–32) but not irrelevant oligonucleotide OCT-1 (Fig 4A, lanes 14, 21 and 28) successfully competed-out binding of ERα to specific ERE-containing oligonucleotides. In conclusion, our results demonstrate the ability of the MUC1 promoter EREs to specifically bind ERα *in vitro*. Further experiments will be needed to study the interaction between ERα and MUC1 specific EREs *in vivo*.

## Discussion

The expression of multiple isoforms of the MUC1 gene [2,4,7,9] in a variety of cell types [2-6] and the active role of the gene in different physiological processes [9] suggest a fine-tuned transcriptional regulation. Some specific questions that we have addressed in this study include: 1) Does the MUC1 promoter regulate differential expression of isoform specific mRNAs? 2) Is the expression of human MUC1 gene transfected into heterologous (mouse) cells relevant to its endogenous expression in homologous (human) cells? 3) How does estrogen regulate MUC1 transcription? Some of these questions have been answered in this study, others are still issues for our ongoing investigations.

Numerous overlapping and densely distributed *cis*-elements that potentially could bind a multitude of transcription factors demonstrate the structural and functional complexity of the MUC1 promoter. Further evidence of the MUC1 promoter complexity is the presence of several elements that participate in the development of RNA-Pol II initiation complexes (TATA-boxes, GC-boxes and initiators) and multiple transcription start sites (CAP-sites).

Usually, eukaryotic promoters that regulate transcription by RNA polymerase II belong to one of four types: 1) TATA-box-containing promoter (facultative genes) [21]; 2) GC-box-containing promoter (housekeeping genes) [22]; 3) Initiator-containing promoter (first described in genes expressed in B-cells) [23]; 4) "Dual" promoter that have both TATA- and GC-elements and drive both TATA- and GC-boxes specific transcription (found in cathepsin D and methallotheonin genes) [27]. The MUC1 promoter demonstrates properties characteristic for promoters of different types. It contains several TATA- and GC-boxes and a number of initiators (see "Additional files 1, 2, 3"). It contains multiple *cis*-elements that potentially transcribe the MUC1 gene in many different cell types and tissues and *cis*-elements specific for viral promoters [2,3,5,28-30]. These peculiarities of the MUC1 promoter allow us to classify it as a "mixed polypotent promoter". We believe that studies of eukaryotic transcription will reveal increasing number of genes with this type of promoter.

Our bioinformatic study of the MUC1 promoter resulted in construction of the MUC1 *cis*-element map. This map has several advantages. Using the map, one may design a functional analysis of transcription factors potentially competing for overlapping *cis*-elements in the MUC1 promoter. It also allows prediction of cells in which the MUC1 gene might be expressed. Based on the content of the MUC1 *cis*-elements, we predicted expression of the MUC1 gene in lymphoid and muscle cells that had been thought to be MUC1 non-producing cells. Subsequently, the MUC1 expression in these cells was confirmed experimentally [5]. Obviously, in a single study it is impossible to evaluate functional activities of all *cis*-elements detected in the MUC1 promoter. Nevertheless, several *cis*-elements that were identified by our bioinformatic approach (MZF-1, Sp1 and STAT) have been already found active in transcriptional regulation of the MUC1 gene *in vivo *[28-30].

Transient transfection of plasmids that have different deletions within the promoter DNA can identify promoter regions necessary for the expression of specific mRNA isoforms. We combined this approach with our bioinformatics information to experimentally characterize the MUC1 promoter activity. In our study, the Dpr plasmid that contained a full-length MUC1 promoter supported expression only of the MUC1/SEC isoform (Fig. 1B, lane – 1). In contrast, the plasmid DprΔ2154 lacking a ~2 kb fragment deleted from the 5'-end of the promoter demonstrated higher activity and supported expression of both MUC1/SEC and MUC1/TM mRNA (Fig. 1B, lanes – 4 and 5, respectively), indicating that within the deleted fragment are transcriptional repressor binding sites. Our *cis*-element map supports this hypothesis, since the deleted fragment of the MUC1 promoter (-2872/-718) contains multiple binding sites for transcriptional repressors: 9 sites for δEF1-repressor (-2180/-2170, -1546/-1536, -1522/-1512, -1486/-1476, -1462/-1452, -1418/-1408, -1175/-1165, -1029/-1019 and -1008/-998), 7 sites specific for the Gfi-1 repressor (-2627/-2515, -2589/-2574, -2530/-2515, -1738/-1723, -1435/-1420, -1306/-1291 and -1141/-1126), 5 sites specific for YY1-repressor (-2786/-2776, -2772/-2762, -1428/-1418, -1395/-1385 and 1203-1193) and a single ELP repressor binding site (-1172/-1165) (see "Additional files 1, 2").

Plasmid DprΔ2446, lacking an additional 292 bp, elevates MUC1/TM expression but leads to a decrease in the expression of the MUC1/SEC mRNA compared to DprΔ2154 (Fig 1B, lanes – 7 and 8). These observations suggest that the deleted 292 bp fragment might contain both additional sites for MUC1/TM mRNA repressors as well as sites important for MUC1/SEC expression. Indeed, as the map shows, the deleted fragment contains *cis-*elements specific for two repressors, YY1- (-670/-660) and δEF1- (-472/-462), in addition to several *cis*-elements specific for mammary epithelial cell activators: CTF/NF, ESE1, MAF, MGF, MP4 and RME [31-34] (see "Additional file 3").

According to results obtained with the DprΔ2839 plasmid, a minimal set of transcription elements (one TATA-box, one CAP-site and several *cis*-elements specific for transcriptional activators RCE, SRE and ETF-RE) (see "Additional file 3") is sufficient for expression of MUC1/SEC isoform (Fig. 1B, lane – 10). Transcriptional activity of the minimal MUC1 promoter has been observed in transfection studies [35,36]. Notably, two very different plasmids, one of which contains the full MUC1 promoter (Dpr) and the other which contains only a minimal promoter sequence (DprΔ2839), drive similar expression of the MUC1/SEC isoform. It appears that in our experimental conditions, the positive and negative effects of the *cis*-elements in the full promoter seem to balance-out each other resulting in expression of MUC1/SEC similar to that of the minimal promoter.

The results demonstrating higher activity of the truncated promoter (DprΔ2154, DprΔ2446) compared to the full length MUC1 promoter (Dpr) are not surprising. Earlier, Kovaric et al [36] showed that expression of the CAT-test gene driven by the full length MUC1 promoter was almost half than that directed by 743 bp promoter fragment. Abe and Kufe [35] also observed higher CAT activity when the test-gene was driven by a smaller promoter fragment (-686/+33) than by a larger one (-1656/+33). Moreover, according to these authors, the 114 bp fragment of the MUC1 promoter located between -598 and -485 bp demonstrated the highest CAT gene expression. Superimposition of the promoter fragments analyzed in these studies and our MUC1 *cis-*element map leads to similar schematics of activating and repressing regions.

One of the issues addressed in our research was whether the expression of the human MUC1 gene transfected into heterologous (mouse) cells is relevant to its endogenous expression in homologous (human) cells. The results described in this study showed that in our experimental conditions, the patterns of the MUC1 gene expression in both cell systems were different. In mouse cells, human MUC1 gene (Dpr plasmid) directed expression of the MUC1/SEC isoform, whereas in human cells, the predominant mRNA was MUC1/TM. In transfected mouse DA3 cells, we could not detect human MUC1/Y isoform, whereas in human T47D and MCF7 cells this isoform was observed. The basis for such differential expression is presently being studied.

One of the important issues in regulation of MUC1 expression is the role of estrogen and estrogen receptors. Several studies have showed that the MUC1 gene positively responds to estrogen [16-19]. However, in our study, we observed that not all MUC1 isoforms responded to estrogen in the same manner. The MUC1/SEC mRNA was expressed in T47D cells (ER+ clone 10) only after treatment with estrogen. In contrast to T47D cells, in ER-positive MCF7 cells, expression of the MUC1/SEC mRNA was observed in the absence of estrogen, although, similarly to T47D cells, estrogen increased and 4-OHT decreased its expression. The MUC1/TM mRNA could be expressed both in T47D estrogen receptor positive cells (clone 10) and estrogen receptor negative cells (clone 8) as well as in MCF7 cells. Moreover, this expression was not affected by 4-OHT. The dependence of the MUC1/Y isoform expression on estrogen and ER was not clear cut. On one hand, we observed some increase in MUC1/Y expression in T47D and MCF7 cells after estrogen treatment but on the other hand, 4-OHT did not inhibit its expression. Further experiments will be needed to clarify this matter.

The above data suggest that the expression of MUC1 isoforms in MCF7 cells somehow differs from their expression in T47D cells. Perhaps relevant to our observations is that MCF7 and T47D cells express different levels of steroid receptors. In MCF7 cells, ER is expressed at much higher levels than progesterone receptors (PR), whereas in T47D cells, the expression of PR is higher than that of ER [37,38]. Since estrogen regulates the transcription of the ER gene [39], it appears that T47D cells may require exogenous supplements of estrogen to activate expression of the ER gene. In contrast, the endogenous expression levels of estrogen receptors in MCF7 cells might be high enough to support expression of the MUC1/SEC isoform. In accordance with our results, Hurd et al [40] observed expression of hyperphosphorylated retinoblastoma protein (ppRB) in MCF7 but not in T47D cells when cells incubated without estrogen. A gradual increase of its expression after treatment with estrogen was observed in MCF7 cells in time-dependent manner. In T47D cells, longer estrogen treatment was necessary to detect ppRB than in MCF7 cells.

Our data demonstrating the responsiveness of the MUC1/SEC isoform expression in human epithelial cells to inhibitory effects of 4-OHT are in agreement with observations that the expression of MUC1 gene in human adenocarcinoma cells is also sensitive to antiestrogens [41,42]. These data suggest that, in human cells, estrogen may regulate the MUC1 gene transcription by interaction with ER directing them to *cis-*ERE in MUC1 promoter. The results obtained by us with EMSA using T47D cell lysates support this hypothesis. However, they are in contradiction with the observations made in a mouse system. Studying the role of ER in transcriptional regulation of the mouse Muc1 gene, Zhou et al [18] concluded that ER did not directly bind to the *cis-*ERE of the murine Muc1 promoter. We found that all *cis*-ERE detected in the human MUC1 promoter could form complexes with human ERα *in vitro*. Several factors may explain this discrepancy. First, different *cis*-EREs were used in both studies. Although human and mouse MUC1 promoters have high degree of homology, their *cis*-EREs demonstrate pronounced diversity. We have analyzed the mouse Muc1 and human MUC1 promoter sequences and found that each promoter contains six *cis*-EREs. Comparison of these elements revealed both homology and differences in their sequences. Second, Zhou et al [18] analyzed binding of estrogen receptors that have been *in vitro *translated, whereas in our binding assays we used ERα endogenously synthesized in T47D cells that express human MUC1 gene.

It should be noted, that, although all MUC1 *cis*-EREs bound ERα, the properties of the complexes developed by different *cis*-EREs were different. Several *cis*-EREs, (ERE1, ERE3, ERE4 and ERE5) containing only half of the classical palindrome sequence produced a weak, fast migrating complex with ERα. Interestingly, ERE2, which also contains only half of the ERE palindrome sequence, developed two complexes that at least partially correspond to those observed with classical ERE from the vitellogenin gene and with "*putative*" ERE-6 of the MUC1 gene. The different electrophoretic mobility of the complexes might be explained by content of ER-cofactors in the complexes. It is not clear why oligonucleotides that have identical or very similar ERE core sequences recruit different cofactors to ER-containing complexes, however, the flanking sequences may play a crucial role in this process [43]. Importantly, all complexes developed with tested *cis*-ERE contained ERα since the binding of ERα was specific and could be inhibited by antibodies developed against the DNA-binding domain of human ERα.

Whereas our *in vitro *studies clearly showed that physical binding of ERα with *cis-*EREs of the MUC1 promoter occurs, definitive *in vivo *binding still remains to be proven. Orientation of ERE within the synthetic oligonucleotides appears not to be important for *in vitro *binding, but orientation and distribution of EREs among *cis*-elements within promoter, are presumably crucial for proper *in vivo *binding [44-46]. A special approach will be needed to study this issue. In the MUC1 promoter, two estrogen responsive elements (ERE-1 and the "putative" ERE-6), have direct sequence orientation while others (ERE-2, ERE-3, ERE-4 and ERE-5) have opposite orientation. Three elements (ERE-1, ERE-2 and ERE-3) are located relatively distant from the active TATA-box and from each other. Three others (ERE-4, ERE-5 and the "putative" ERE-6) are located in close proximity to each other between nucleotides -389 and -337 (see "Additional files 1, 2, 3") and may function as an estrogen responsive unit [47,48].

Very few mRNAs that are known to be directly regulated by estrogen in mammary epithelial cells are actually induced via canonical EREs [49]. In fact, most estrogen-responsive genes identified to date contain one or more imperfect EREs or multiple copies of an ERE half-site rather than the classical ERE [50,51]. The MUC1 *cis*-EREs also are not absolutely identical to the consensus sequence. Although the affinity of the estrogen receptor for the classical ERE is higher than for imperfect ERE-like sequences, most of the imperfect EREs bind ER [52]. The MUC1 *cis*-EREs, although imperfect, also bound ER *in vitro*. It is becoming clear that EREs function as allosteric modulators of ER conformation [53,54]. The conformational changes in ER induced by individual ERE sequences lead to specific association of the receptor with other transcription factors and assist in differential transcription of estrogen-responsive genes [54,55]. In light of these data, the imperfect MUC1 *cis*-EREs might be significant, perhaps by differential usage of ERE in diverse cells recruiting distinct cofactors for the MUC1 expression.

We have discussed the possible involvement of ER and MUC1 EREs in regulation of the MUC1 gene transcription. However, it is known that ER may regulate gene transcription also by interaction with other transcription factors (STAT, AP1, EGFR or NFkB) without direct binding to ERE [56]. Further studies are needed to understand the process of MUC1 transcription *in vivo *and to elucidate the mechanism by which estrogen activates transcription of the MUC1 gene.

Although our study revealed some new and important features of the MUC1 promoter *cis*-element content and structure, the precise mechanisms by which these *cis*-sites are involved in the regulation of MUC1 expression have not been fully elucidated. On one hand, elements within the promoter could determine usage of different transcription start sites specific for individual MUC1 isoforms. The presence of multiple CAP-sites in the MUC1 promoter together with previously documented multiple transcription start sites of the MUC1 gene in T47D cells [11] support this hypothesis. On the other hand, the promoter *cis*-elements might be involved in regulating of alternative splicing of a single pre-mRNA common to all MUC1 isoforms. A growing body of evidence suggests that transcription and splicing are highly coordinated processes both at the structural and functional levels [57-61]. For instance, it has been shown that mutations introduced into promoter *cis*-elements could change the alternative splicing patterns [58]. Moreover, the RNA pol II large subunit physically associates with spliceosomes and the SR proteins that regulate alternative splicing act through specific promoter occupation [62].

In light of these data, we suggest that *cis*-elements of the MUC1 promoter may be involved in mechanisms that regulate both transcription and splicing of the MUC1 pre-mRNAs. In this study, we used total RNA extracted from transfected cells. However, for a better understanding of the role of the MUC1 promoter in transcription and splicing of MUC1 isoforms, an additional study of the 5'-ends of nuclear pre-mRNA is needed. Additionally, the effect of different mutations within *cis*-sites of the MUC1 promoter on isoform expression could be more thoroughly dissected. These experiments are currently in progress.

## Conclusion

In this study several important findings have been revealed. First, transcription *cis*-elements of the MUC1 promoter have been identified and the map of these elements has been constructed. The advantage of such a map is that it not only shows the content of the promoter *cis*-elements but exemplifies the transcription factors potentially competing for specific binding sites. Second, we established that different regions of the MUC1 promoter may control expression of different MUC1 isoform RNAs. We also observed differential expression patterns of the human MUC1 gene in heterologous (mouse) and homologous (human) cells. Third, we showed, for the first time, that estrogen can differentially regulate the expression of some MUC1 isoforms. For instance, estrogen activated expression of MUC1/SEC, but not MUC1/TM isoform in human breast cancer epithelial cells. Fourth, we found six *cis*-ERE in the MUC1 promoter and showed that they can bind ERα *in vitro*. Our findings may help to develop molecular modalities for controlled regulation of the MUC1 gene expression and thus may contribute to progress in molecular-based breast cancer therapy.

## Methods

### Computer analysis of potential transcription cis-elements

The computer analysis of the potential transcription *cis*-elements of the MUC1 promoter has been performed by using the TRANSFAC (MatInspector V2.2) and TSSG databases of transcription factors and their DNA binding sites [63,64]. The 2.9 kb MUC1 promoter sequence was obtained from the GENE Bank NCBI: accession #-X69118. For construction of the MUC1 promoter *cis*-element map we used mainly those *cis*-elements whose sequence homology with corresponding consensus sequences was significantly high (0.75–1.0).

### Cells

Human breast cancer cell lines MCF-7, T47D and MDA-231 and mouse breast cancer DA3 cells were cultured in full DMEM medium with 10% foetal calf serum (FCS), penicillin (100 u/ml), streptomycin (100 μg/ml) at 37°C in 5% CO_2_. One week before estrogen treatment, cells were transferred to phenol red-free DMEM medium containing antibiotics and 5% charcoal-treated FCS. The concentration of FCS was reduced to 1% one day before adding of 0.1–10 nM 17β-estradiol (E2) only or in combination with 10 nM – 1 μM 4-hydroxytamoxifen (4-OHT) (Sigma). Cells were incubated for 24 hours and then were harvested for RNA preparation.

### Plasmids

The plasmid Dpr (pDpr) containing the human MUC1 full-length promoter and MUC1 coding sequence was constructed on a polyIII plasmid backbone [65]. The genomic MUC1 sequence was inserted into ppolyIII at the *Eco*RI site. The recombinant plasmid was then digested with restriction enzyme *Pvu*I generating two fragments of 6946 bp and 666 bp. The bigger fragment contained a part of ppolyIII, the full MUC1 promoter sequence (2872 bp) and all MUC1 exons and introns except for a part of cytoplasmic domain coding DNA. This DNA fragment together with 150 bp SV40 polyA sequence and 600 bp of the ppolyIII was isolated from the pCL642/MUC1/TM by *Pvu*I digestion [11]. The obtained *Pvu*I/*Pvu*I 870 bp fragment was ligated with 6946 bp *PvuI*/*Pvu*I fragment resulting in generation of pDpr. This plasmid contained the full-length promoter and the full coding region of the MUC1 gene. It was used for generation of deletions in the promoter region (Fig. 1A). The first deletion mutant of the MUC1 promoter was constructed by excision of 2154 bp promoter region from the pDpr by *Sac*I digestion followed by self-ligation of the rest of the pDpr. The resultant plasmid, pDprΔ2154, lacks the 5'-end 2154 bp promoter fragment. The next deletion mutant designated pDprΔ2446 represented the pDpr without 5'-end 2446 bp promoter sequence. The promoter region of the pDprΔ2446 is 292 bp shorter at the 5'-end than promoter fragment of the pDprΔ2154. The pDprΔ2446 was generated by *Sac*I/*Age*I excision of almost full MUC1 promoter followed by insertion into the rest of pDpr sequence of the PCR product that covered the region between -426 and -33 nucleotides of the MUC1 promoter. The primers used for synthesis of the insert were:

5' AATCTA*GAGCTC*GCTCTGCTTCAGTGG 3' (forward) (the underlined sequence is SacI restriction site)

5' GCTTTAT*ACCGGT*CCCCCCACTCCCCG 3' (reverse) (the underlined sequence is AgeI restriction site)

The third promoter deletion mutant, pDprΔ2839, contained only 33 bp MUC1 promoter sequence (-33/+1) and included the TATA-box and several transcription *cis*-elements. It was constructed by excision of the 5' 2839 bp from the pDpr by *Sac*I/*Age*I digestion and ligation of the rest of the pDpr with the *Sac*I-*Age*I adapter.

### Transient transfection assay

Transient transfection assays were performed in 60 mm Petri dishes. Plasmid DNA (5 μg) was transfected into DA3 cells by Lipofectin as described by the manufacturer. Efficiency of transfection was evaluated by simultaneous transfection of GFP-expression plasmid. After 48 hours cells were harvested for RNA preparation and RT-PCR analysis.

### RT-PCR

Total RNA was isolated from the cells using TRIzol Reagent (Gibco BRL). cDNA was synthesized by EZ-First Strand cDNA Synthesis Kit for RT-PCR (Biological Industries, Beit Haemek Ltd, Israel) according to the manufacturer. The complementary DNA products from RT reactions were amplified by PCR in the presence of the appropriate primers:

MUC1/SEC (forward): 5' TTCCCAGCCACCACTCTGATACT 3'

MUC1/SEC (reverse): 5' AGCATGGGGAAGGAAAGG 3'

MUC1/TM (forward): 5' TTCCCAGCCACCACTCTGATACT 3'

MUC1/TM (reverse): 5' GACATTGATGGTACCTTCTCG 3'

MUC1/Y (forward): 5' GTATAAAACGGAAGCAGCCTCTC 3'

MUC1/Y (reverse): 5' GAGAGGCTGCTTCCGTTTTATAC 3'

Actin (forward): 5' GTTTGAGACCTTCAACACCCC 3'

Actin (reverse): 5' GTGGCCATCTCTTGCTCGAAGTC 3'

The MUC1 and actin specific fragments were amplified using the following PCR conditions: denaturation at 94°C for 5 min, then 35 cycles of 94°C for 45 sec, 50°C for 45 sec and 72°C for 45 sec followed by final primer extention of 72°C for 5 min. All transcripts were analyzed in parallel on at least three separate occasions in a thermal cycler. PCR products were analyzed by electrophoresis on 1.2% agarose gel and visualized by ethidium bromide staining under UV illumination.

### Electrophoretic Mobility Shift Assay (EMSA)

EMSA was carried out as described by Promega Protocol [66]. The following oligonucleotides corresponding to ERE present in MUC1 promoter (the bold underlined sequence) in direct (ERE-1 and ERE-6) and inverted (ERE-2, ERE-3, ERE-4 and ERE-5) orientation were annealed with corresponding complementary oligonucleotides and end-labeled with [γ-^32^P]ATP using T4 polynucleotide kinase.

ERE-1 -2754 5' AGAGGGT***GGTCA***CCCACCCTTC 3'-2733

ERE-2 -1182 5' TTGAACCCC***TGACC***TTGTGATCC 3'-1160

ERE-2 mut-1182 5' TTGAACCCC**A**GAC**A**TTGTGATCC 3'-1160

ERE-3 -755 5' AAGGCTCCCGG***TGACC***ACTAGAG 3'-736

ERE-4 -389 5' AGTGGG***AGACC***TAGGGGTGGG 3' -369

ERE-5 -371 5' GGGCTTCC***CGACC***TTGCTGTA 3'-351

ERE-6 ("*putative*") -351 5' TACA***GGACC****T****CGACC***TAGCTG 3' -332

ERE-6 mut -351 5'TACA***A****GA****TT****TCG****GA****C*TAGCTG 3'-332

Vit-ERE 5'TCCA***GGTCA****CTG****TGACC***CAAC 3'

Vit-EREmut 5'TCCA*GG****A****C****T****CTGT****A****AC****G***CAAC 3'

Cell nuclear extract was prepared as described [67]. Nuclear extracts (2–5 μg) were incubated in 1 × Gel Shift Binding Buffer (50 mM Tris, pH 7.5/4% glycerol/1 mM MgCl_2_/0.5 mM EDTA/0.5 mM DTT/50 mM NaCl/0.05 mg/ml poly(dI-dC)poly(dI-dC) at room temperature for 10 minutes, and then ^32^P-labeled oligonucleotides (80,000 cpm) were added and incubation at room temperature was continued for 20 minutes. The anti-ERα antibody [anti-ERα Ab (D-12), catalog # x:sc-8005X, Santa Cruz Biotechnology] used in the study were raised against DNA-binding domain of human ERα (amino acids 2–185 mapping at the N-terminus). Incubation of the binding reaction mixture with anti-ERα Ab was performed for 1 hr at room temperature or overnight at 4°C before adding labeled ("hot") oligonucleotides. Competing non-labeled ("cold") oligonucleotides, or non-relevant OCT1 oligonucleotide were added to reactions simultaneously with "hot" ^32^P-ERE oligonucleotide probe. 1 μl of 10× loading buffer was added per reaction to stop binding and reaction products were analyzed by electrophoresis in 6% DNA retardation gel in 0.5 × TBE buffer at 290 V. Gel was dried on a gel dryer and exposed to Kodak X-ray film at -70°C with an intensifying screen.

### Statistics

All transfection assays and expression PCR analysis as well as EMSA experiments were performed in parallel on at least three separate occasions.

## Abbreviation

All abbreviation of transcription factors and corresponding *cis*-elements are from [63,64].

## Competing interests

We think that there may be competing interests between our group and Dr. S. Gendler and Dr. D. Carson groups. Studying the role of estrogen receptors (ER) in transcriptional regulation of the mouse *MUC1 *gene, Zhou X, DeSouza MM, Julian J, Gendler SJ and Carson DD (*Mol Cell Endocrinol *1998, 143:65–78) concluded that ER did not bind directly to the *cis*-ERE in murine *MUC1 *promoter. In contrast to the results obtained by these authors in mouse system, we found that all *cis*-ERE detected in human MUC1 promoter could form complexes with human ERα *in vitro*. Several factors that may explain this discrepancy are discussed in our manuscript.

## Authors' contributions

JZZ designed the study, constructed the MUC1 promoter *cis*-element map, performed transfection assays, PCR analysis of MUC1 expression in transfected cells and in human epithelial cells treated with estrogen and 4-OHT, EMSA experiments and drafted the manuscript. IB performed ^32^P-labeled oligonucleotides, part of EMSA experiments depicted in Fig. 4 and participated in computer analysis of the MUC1 promoter. YA generated plasmids pDprΔ2154, pDprΔ2446 and pDprΔ2839 and participated in transfection assays. MG performed part of computer analysis of the MUC1 promoter *cis*-elements. DHW prepared pDpr plasmid, participated in design of the study and analyzing of the results. IK designed part of the study, performed T47D cell analysis, selected clones 8 and 10, participated in experiments depicted in Fig. 3. All authors read and approved the manuscript.

## Supplementary Material

Additional file 1 **The map of transcription factor *cis*-elements present in the MUC1 promoter**. The file contains part of the "map" that represents promoter sequence -2872/-1913. Click here for file

Additional file 2 **The map of transcription factor *cis*-elements present in the MUC1 promoter**. The file contains part of the "map" that represents promoter sequence -1912/-713 Click here for file

Additional file 3 **The map of transcription factor *cis*-elements present in the MUC1 promoter**. The file contains part of the "map" that represents promoter sequence -712/+69 Click here for file
