# Supplementary figures and images for: MUC1 gene overexpressed in breast cancer: structure and transcriptional activity of the MUC1 promoter and role of estrogen receptor alpha (ERα) in regulation of the MUC1 gene expression
Source: Mol Cancer. 2006 Nov 5;5:57. doi: 10.1186/1476-4598-5-57 (PMC1636664; doi:10.1186/1476-4598-5-57)

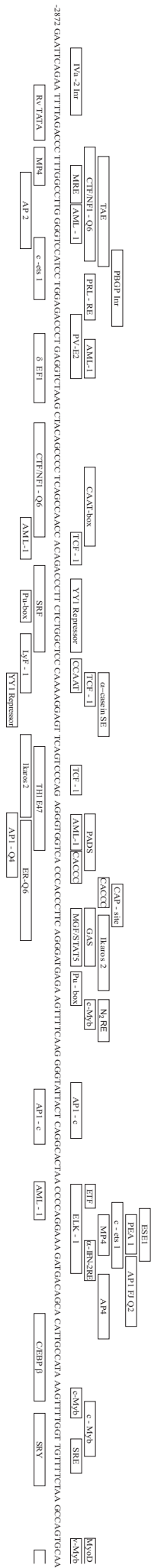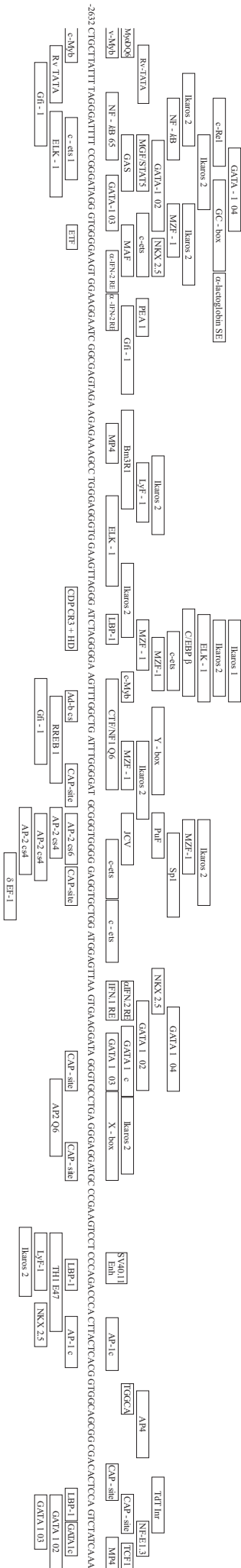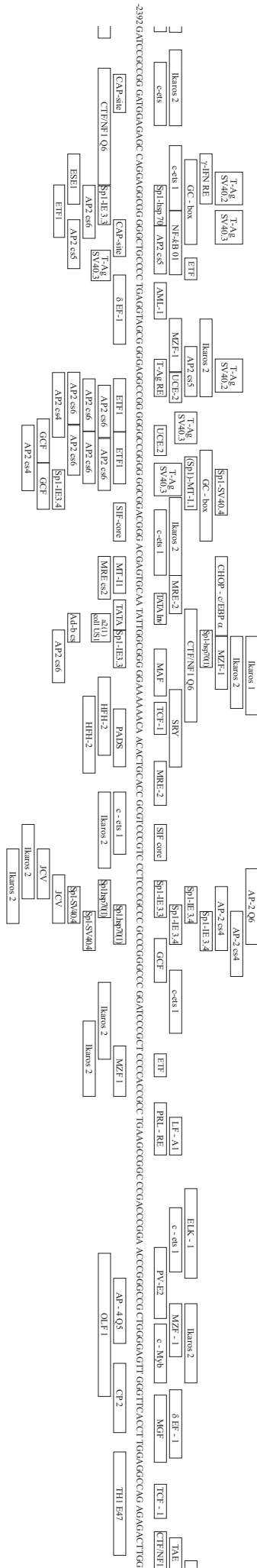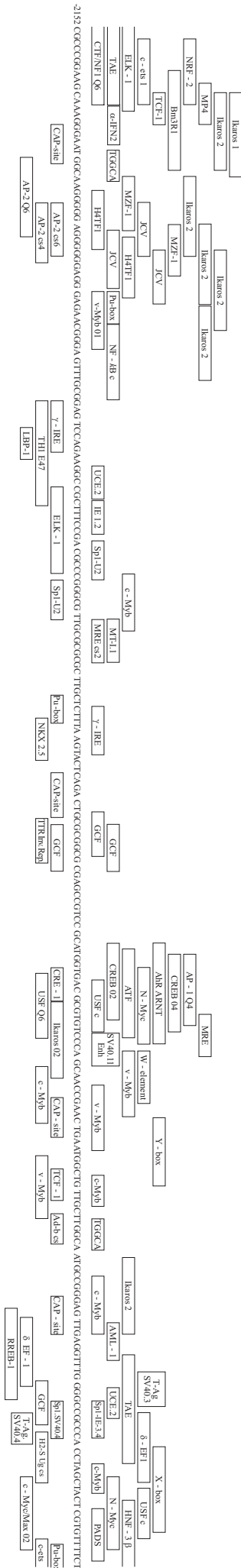

Supplement: Additional file 1 — The map of transcription factor cis-elements present in the MUC1 promoter. The file contains part of the "map" that represents promoter sequence -2872/-1913. [file 1476-4598-5-57-S1.pdf]

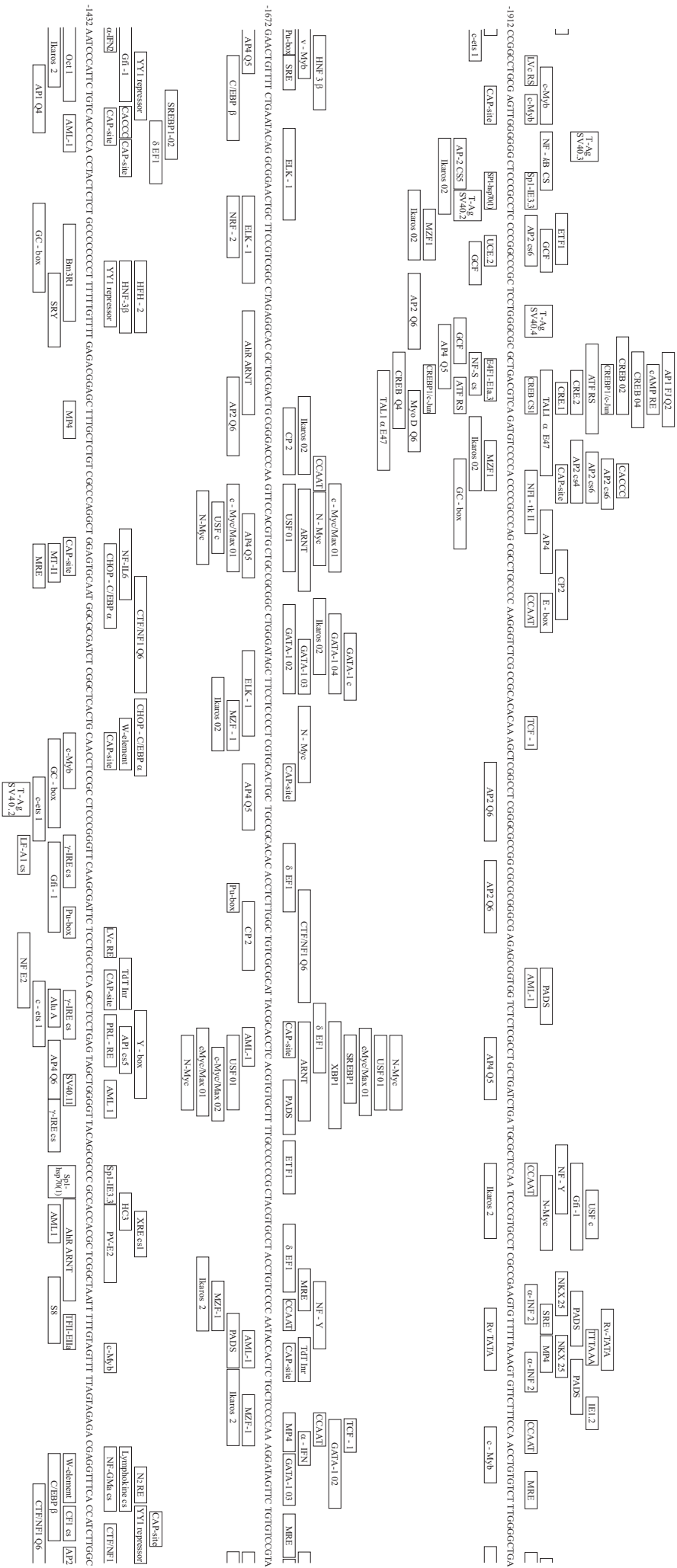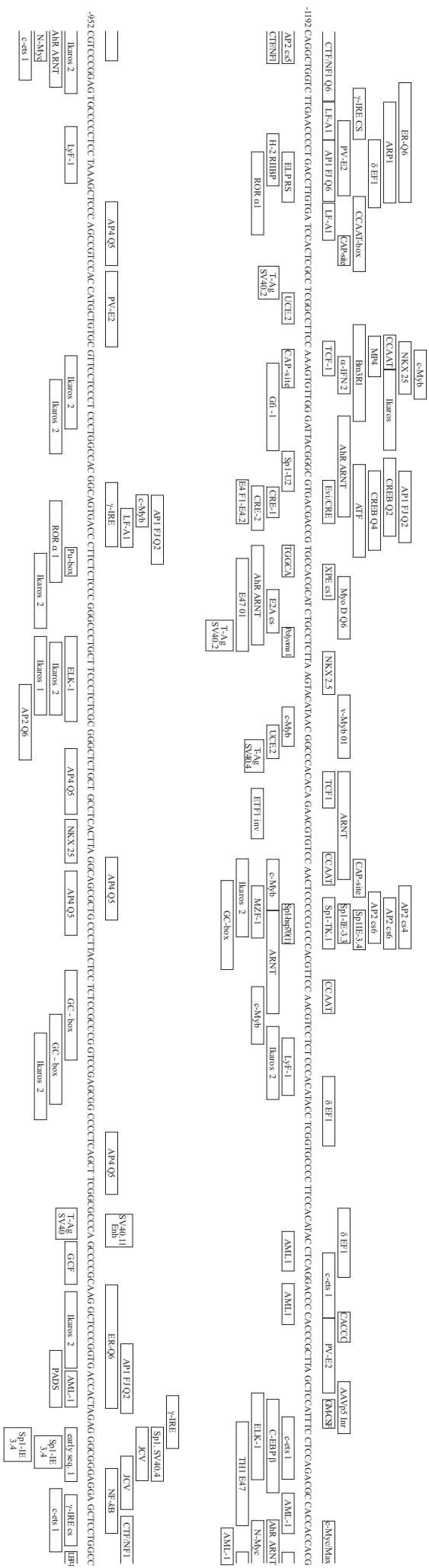

Supplement: Additional file 2 — The map of transcription factor cis-elements present in the MUC1 promoter. The file contains part of the "map" that represents promoter sequence -1912/-713 [file 1476-4598-5-57-S2.pdf]

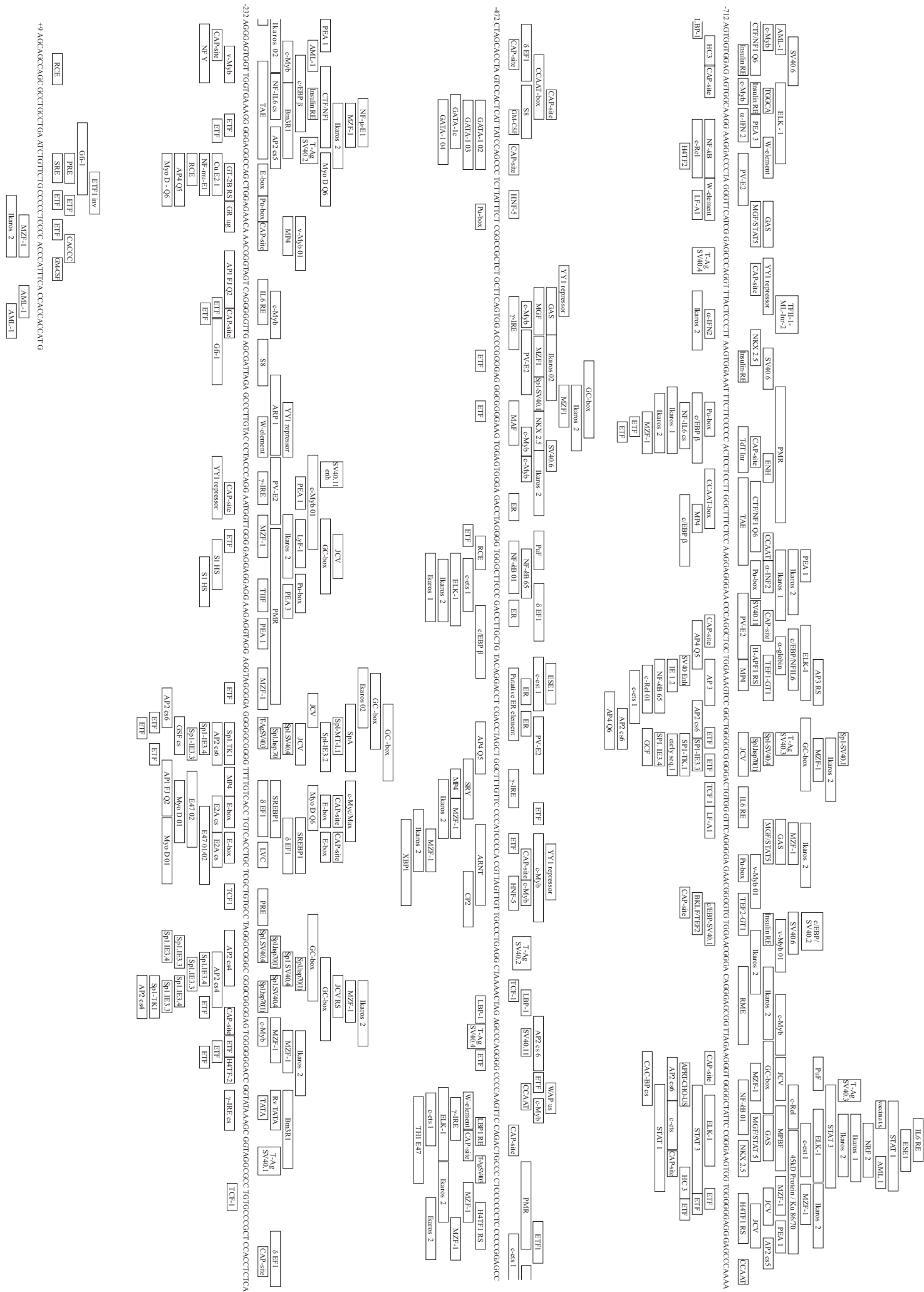

Supplement: Additional file 3 — The map of transcription factor cis-elements present in the MUC1 promoter. The file contains part of the "map" that represents promoter sequence -712/+69 [file 1476-4598-5-57-S3.pdf]
